# Case report on meningoenceplapathy in an Indian patient post CoViD vaccination

**DOI:** 10.6026/97320630019117

**Published:** 2023-01-31

**Authors:** Raj Kamal Choudhry, Amrendra Kumar Singh, Kumar Sourav, Anjum Pervez, Abilesh Kumar, Subham Kumar

**Affiliations:** 1 Jawaharlal Nehru Medical College and Hospital, Bhagalpur, Bihar, India

**Keywords:** COVID-19, COVAXIN, immunization, methyl prednisolone, post vaccine meningoencephalitis

## Abstract

The SARS-COV-2 infection-related severe illness is prevented by vaccinations. Therefore, it is relevant to report a case of post vaccine meningoencephalitis in a 30 year old male Indian patient, who presented with weakness in all the extremities, episodes of loose stool, fever, vomiting, tachypnea and loss of consciousness immediately following the 2nd dose of the COVID vaccination (COVAXIN).

## Background:

Numerous immunizations have been proven to dramatically lower the chance of developing a severe COVID-19 infection, with an excellent benefit/risk ratio and tolerable safety profile. [1] As of September 5, 2021, >5 billion vaccine doses had been given throughout the world. [2] A guarded mass of substantial neurologic unfavorable events following immunization among 1st dose patients was quickly identified by the surveillance system. [3] Meningoencephalitis after getting COVID-19 vaccines, however, is a very uncommon disease. Meningoencephalitis was initially detected after getting the 2nd dosage of the vaccination of Covid-19 Pfizer. It was observed that we should begin administering Acyclovir vial IV 750 mg 3 times a day up to14 days since we suspect viral meningitis [4]. Since it was reported in the case which was held in 2021 [4] that the patient responded to the acyclovir quite well; within two days, she was able to regain consciousness and her sense of orientation, and after 14 days, she was relieved from the hospital with no issues related neurological deficit.

## Case presentation:

A 30-year-old male patient presented to Jawaharlal Nehru medical college, Bhagalpur with a history of sudden headache at 3:00 pm on 30th September 2021, followed by weakness in all the extremities, episodes of loose stool, fever, vomiting, tachypnea and loss of consciousness immediately following the 2nd dose of the COVID vaccination at 11:00 am on 30th September 2021 in a private nursing home. The family members of the patient were saying that he didn't experience this before. So the onset of the illness was after second dose of Covid vaccine. Past medical history was insignificant with no history of Fever and seizure disorder. Symptoms were started from the same day evening of the second dose of Covid vaccine, COVAXIN and it was worsening as the days were passing. The actual palliates are unknown but we can say that post vaccine complications, which are generally fever, body ache and fatigue. Tachypnea and weakness can be provoked by exercise, anxiety, stress and anger. Patient experienced fast breathing and breathlessness for the first time and severe headache, followed by extreme weakness, and unconsciousness. Condition was worsening day by day, only recovery was responding commands with eyes. Upon investigation, it was discovered that the patient was unconscious, confused, and disoriented. Evaluation of acute flaccid paralysis was present thrombocytopenia and seizures were present. Positive symptoms of irritation related to meningeal were seen. There were bilateral is cordial pupils, and the light sensitivity was similarly good. The sign of sepsis was unfavorable. The patient was unable to walk or stand. Head, lung, Chest, stomach and brain were the site of symptomatology. Laboratory investigations revealed agranulocytosis and moderate leukocytosis were detected in the whole blood count with increased C reactive protein (CRP). Renal and liver function tests were a present, along with serum electrolytes. The random blood sugar level was checked. The CT scan of the brain was done. An MRI of the brain was performed after 12 hours. Microscopically, the lumbar puncture revealed lymphocytic pleocytosis. HSV type 1and type 2 viral polymerase chain reactions (PCR) and HIV test results were negative. Based on physical examination and laboratory findings a provisional diagnosis of Post Vaccine Meningoencephalitis was made. Patient was administered with Methyl Prednisolone 1 gm daily and Immunoglobulin. The patient condition improved after 8 months. Recovery was shown as following vocal commands by eye movement. At last, the patient recovered in August 2022.

## Discussion:

The pandemic named COVID19 had a disastrous effect on social life, the economy, and public health. [5] The neurological system is known to be affected, leading to multiple neuropathy, aortic ischemic stroke, and encephalopathy. It had and still does have a huge impact on people's lives on a personal and societal level, leading to psychological issues and adjustments in health behavior. Fast-changing pandemic conditions necessitate creative approaches to continue providing clinical prevention services, such as vaccination, to immunize and in order to prevent over taxing healthcare institutions and eventual systemic collapse. It has been established that the creation of a vaccine is the only method of containing the epidemic. The SARS-COV-2 infection-related severe illness is prevented by vaccinations.[6] The development and delivery of COVID-19 vaccines help to prevent the disease, although it is unclear how long the vaccine-induced immunity lasts until after patients have first received the vaccine. Four main vaccination strategies have been studied for the COVID-19 vaccines: mRNA-based vaccines inactivated virus-based vaccines, DNA-based vaccines, and protein-based vaccines. DNA-based vaccines induce the production of spike proteins by cells by introducing the DNA code in the SARS-CoV-2 spike protein into the cells via viral vectors. Similar to this, mRNA vaccines function by delivering mRNA to cells via lipid nanoparticles. Protein vaccines are constructed using all or part of the spike protein. The SARS-CoV-2 virus is used as an ingredient in several vaccines. The aim of the vaccination is to produce antibodies that can either flag diseases for immune system eradication or neutralize them. [7] There have been instances of neurological side effects like demyelinating episodes and thrombosis related to cerebral sinus occurring after vaccination. In the medical trials of the main vaccines of SARS-CoV-2, there were only a few rare incidences of neurological problems.[8] According to Finsterer et al. there were multiple examples of neurological issues, and it appeared that a recent SARS-CoV-2 vaccine may have contributed to the neurological issues. An elderly woman experienced an ischemic stroke 7 hours after receiving the 1st dose of an mRNA-based vaccination; however it was not obvious whether it was a direct fundamental link between the brain stroke and vaccines. 4 days following the 1st dose of SARS-CoV-2 which is a vector-based immunization, a woman of 29-years-old experienced cerebellar stroke (anischemic).

Eight days following the 1st dose of SARS-CoV-2 immunization-a vector-based, an age of 32-year male who had GBS relapsed. By the end of June 2021, minimum 21 patients with vaccination of SARS-CoV-2 associated GBS had been identified. [9] 7 out of 37,000 vaccine recipients who received the mRNA vaccine had Bell's palsy, according to data from the clinical studies. A case with 3 prior episodes of Bell's palsy was described in < 36 hours following the 2nd dosage of the Pfizer COVID-19 Vaccine. [10] 3 reported cases of myelitis that of transverse, in trials for theChAdOx1nCoV-19 vaccination were examined by an self-governing panel of neurological experts; two were found to be unconnected, and one was thought to be possible. [11] Due to these effects, vaccination studies and roll-out initiatives have been temporarily halted in a number of nations. Programs have been resumed in the absence of strong evidence linking the vaccine to adverse events or the rarity of consequences. Although there were no suspected occurrences of meningoencephalitis according to Biological E. limited vaccine safety reports, it was reported the very first case of rare aseptic meningitis following the first intramuscular injection of covid-19 vaccine of the BNT162b2 mRNA. [12] After receiving the influenza, measles, mumps, and rubella, vaccines, cases of aseptic meningitis have been mentioned. The precise reason of meningitis that develops after vaccination is uncertain. Though, the chance exists that autoimmune meningitis will develop as a result of the molecular mimicry set off by the protein produced during immunization. The blood-brain barrier may be broken by an autoimmune (self-immune) response or the S1 protein formed by the vaccine itself, leading to a septic meningitis. The first instance of meningoencephalitis was documented in the current trial 5 days following the second dosage of the Pfizer-COVID-19 vaccine. Both meningitis and encephalitis, which are infections of the brain and meninges, can have serious fatal effects as well as long-standing neurological effects, especially in young kids in underdeveloped nations. These illnesses are commonly grouped together under the general name acute meningoencephalitis since they are challenging to diagnose clinically.[13] Meningoencephalitis can damage the meninges, grey or white matter, or both, in a localized or widespread manner. There are four main forms of meningoencephalitis. Herpes viruses are the most frequent cause of meningitis and encephalitis in countries of western ghat, while vector (carrier)-borne diseases are the most frequent cause in temperate mounting nations. [14]

## Conclusion: 

We report a case of post vaccine meningoencephalitis in a 30 year old male Indian patient, who presented with weakness in all the extremities, episodes of loose stool, fever, vomiting, tachypnea and loss of consciousness immediately following the 2nd dose of the COVID vaccination (COVAXIN).
